# Effectiveness of potassium peroxymonosulfate against enveloped viruses using an aqueous phase and its application on various contaminated carrier surfaces and artificially avian influenza virus-contaminated clothes

**DOI:** 10.14202/vetworld.2024.2595-2602

**Published:** 2024-11-22

**Authors:** Sakchai Ruenphet, Nutnaree Kunanusont, Darsaniya Punyadarsaniya

**Affiliations:** 1 Department of Animal Biotechnology, Mahanakorn University of Technology, Bangkok, Thailand; 2 Department of Immunology and Virology, Mahanakorn University of Technology, Bangkok, Thailand

**Keywords:** avian influenza virus, disinfectant, Newcastle disease virus, potassium peroxymonosulfate, virucidal

## Abstract

**Background and Aim:**

Potassium peroxymonosulfate (PPMS) is a broad-spectrum disinfectant that oxidizes viral protein capsids. The effectiveness of PPMS in killing viruses depends on several factors, including its concentration, contact time, and present of organic materials. This study evaluated the efficacy of PPMS in an aqueous phase. It also applied PPMS to artificially avian influenza virus (AIV)-contaminated carrier surfaces and clothes and compared its effectiveness with that of sodium dichloroisocyanurate (NaDCC) and quaternary ammonium compounds (QAC).

**Materials and Methods:**

Four PPMS concentrations (1×, 0.5×, 0.25×, and 0.125×), were evaluated for their virucidal efficacy against Newcastle disease virus (NDV) and AIV in an aqueous phase. The evaluation included testing in the absence and presence of organic materials under different exposure times, such as 5 s, 30 s, 1 min, 3 min, 5 min, 10 min, and 15 min. AIV inactivation was assessed on contaminated carrier surfaces, such as stainless steel, rubber, plastic, and artificially contaminated clothes.

**Results:**

In aqueous phase, concentrations of 1×, 0.5×, 0.25×, and 0.125× inactivated NDV in the absence of organic materials within 5 s, 5 s, 5 min, and 15 min at concentrations of 1×, 0.5×, 0.25×, and 0.125×, respectively. In the presence of organic material contamination, NDV could be inactivated within 30 s for 1×, 1 min for 0.5×, and 10 min for 0.25×; however, 0.125× PPMS did not achieve inactivation within 15 min. PPMS concentrations of 1×, 0.5×, 0.25×, and 0.125× inactivated AIV within 5 s, 5 s, 5 s, and 30 s, respectively, in both the absence and presence of organic materials. PPMS at a concentration of 1× could inactivate AIV on all carriers within 30 s. PPMS at 0.5× and 0.25× concentrations could inactivate AIV within 30 s on rubber and plastic; inactivation occurred within 1 min on stainless steel. However, 0.125× PPMS and 1× QAC could not achieve inactivation within 3 min on all carriers. Finally, PPMS concentrations of 1×, 0.5×, 0.25×, and 0.125× inactivated AIV on rayon sheets within 5 s, 30 s, 5 min, and 15 min, respectively. However, the recommended NaDCC concentration achieved inactivation within 10 min, whereas QAC did not achieve inactivation within 15 min.

**Conclusion:**

PPMS can inactivate enveloped viruses such as NDV and AIV. Furthermore, PPMS is superior to NaDCC and QAC for inactivating viruses on various carrier surfaces and artificially contaminated clothes. However, the virucidal efficacy of PPMS depends on the optimal concentration, organic material conditions, and exposure/contact timing. Therefore, PPMS is a promising alternative disinfectant crucial for enhancing biosecurity and controlling viruses that contaminate animal farms, slaughterhouses, and hospitals.

## Introduction

Food products from animals, such as eggs, meat, and milk, are susceptible to contamination by various pathogens, leading to foodborne illnesses such as salmonellosis, colibacillosis, and campylobacteriosis [1]. These diseases often result from specific transmission pathways in poultry farming. Bacterial transmission within chicken products primarily occurs through the transport of cages containing fecal contamination, highlighting the critical role of biosecurity measures in preventing such transmission [2]. Racicot *et al*. [3] have detailed how *Salmonella enteritis* spreads through direct contact, including transmission through infected animals and fomites, such as plastic chicken transport cages. This understanding is crucial for managing both bacterial and viral diseases in poultry. In addition to bacterial infections, viral diseases such as Newcastle disease (ND) and avian influenza (AI) pose significant challenges due to their contagious nature and economic impact on the poultry industry [4]. Infected birds release substantial viral agents from their respiratory and digestive systems, contributing to environmental contamination and worsening economic losses in the poultry sector [5]. Effective disease control requires rigorous implementation of comprehensive sanitation and biosecurity measures on and around farms to mitigate the spread of salmonella and AI. To achieve successful disease control against viral infections, optimizing disinfectants as part of robust sanitation and biosecurity programs for poultry farms is essential. In Japan, the Ministry of Agriculture, Forestry, and Fisheries has implemented a hazard analysis and critical control point (HACCP) system on animal farms, which is integral to ensuring food safety and enhancing disease control measures [6]. In parallel, the Japan Good Agricultural Practice (JGAP), established by the Japan GAP Foundation in 2017, complements the HACCP system by monitoring and regulating animal farm standards to strengthen biosecurity and disease control measures [7]. Enforcement of rigorous biosecurity measures is essential for effectively implementing the HACCP and JGAP systems, which are crucial for maintaining food safety standards and disease control on farms. A vital component of the HACCP strategy is the strategic use of disinfectants to bolster biosecurity measures and mitigate disease risk on and around animal farms.

Bloomfield *et al*. [8] have found that bacteria and virus pathogens present in organic matter from infected birds, such as feces, saliva, and vomitus, exhibit high tolerance to various disinfectants. Due to their resilience to disinfectants, these pathogens can spread through footwear worn by farmers, employees, and farm visitors [3]. In addition, these pathogens can persist on fomites such as food, plastic egg trays, chicken cages, and farm clothes [9–11]. Therefore, effective disease prevention and control, especially in the poultry industry [12], relies on the rigorous application of disinfectants to prevent pathogen transmission through different media.

Potassium peroxymonosulfate (PPMS), also known as potassium monopersulfate (PMPS), is a broad-spectrum disinfectant that oxidizes bacterial and viral protein capsids. This oxidation process leads to the release and inactivation of viral nucleic acids. The effectiveness of PPMS in killing bacteria and viruses depends on several factors, including its concentration, contact time, and presence of organic materials [13]. Due to its safety and versatility, PPMS is widely used as a multipurpose virucidal disinfectant at specific concentrations [6, 14]. For example, it is employed in the livestock industry to disinfect animal shelters, meat production facilities, and swimming pools [15]. In addition, PPMS-containing products deactivate severe acute respiratory syndrome coronavirus, similar to sodium hypochlorite [16]. Although PPMS powder can cause severe skin and ocular burns on direct contact due to its corrosive nature, the solution is safe and non-irritating for animals and humans [6, 15].

This study assessed the efficacy of PPMS against enveloped viruses, including ND virus (NDV) and AI virus (AIV), under varying concentrations, organic material conditions, and exposure times. In addition, the present study examined their application on artificially contaminated carrier surfaces and clothes to enhance biosecurity to control viruses that contaminate animal farms, slaughterhouses, and hospitals.

## Materials and Methods

### Ethical approval

The present study used chicken embryo fibroblasts prepared from 9-day-old embryonic eggs. As the embryos were at an early development stage, ethical approval was not required for this study according to standard ethical guidelines for research on embryonic tissue.

### Study period and location

This study was conducted from December 2019 to May 2020 at biosecurity level-2 facilities of Virology and Molecular Diagnostic Laboratory, Faculty of Veterinary Medicine, Mahanakorn University of Technology, Bangkok, Thailand.

### Sample preparation

PPMS powder (BIOX^®^, Biogénesis Bagó, Argentina) was freshly dissolved in distilled water (dW2) at varying concentrations, including the manufacturer’s recommended concentration of 1% (1×), followed by 0.5% (0.5×), 0.25% (0.25×), and 0.125% (0.125×). To simulate a 5% organic material condition, 500 μL of fetal bovine serum (FBS) was added to 10 ml of each PPMS concentration before testing. The pH of all concentrations was measured before and after neutralization with a blocking solution using a pH meter.

### Viruses and cells

Virulent NDV (NDV/Chicken/Asean Country/2013 [17, 18]) and low pathogenic AIV (A/duck/Aomori/Japan/395/2004 H7N1) were propagated using 9-day-old chicken embryonic eggs, as described by Jahangir *et al*. [19]. Three days post-inoculation, allantoic fluid was collected and stored at –80°C until testing. Chicken embryo fibroblasts (CEF) and Madin-Darby canine kidney (MDCK) cells were then prepared for NDV and AIV titration, respectively [19].

### Blocking solution preparation

A mixture of 1M Tris-HCl (pH 7.4) and FBS at a ratio of 7:3 was used, following the method described by Kunanusont *et al*. [9].

### Virucidal efficacy in aqueous phase

Four hundred microliters of each PPMS concentration were mixed with 100 μL of either NDV or AIV and incubated at room temperature (25°C) for the following durations: 5 s, 30 s, 1 min, 3 min, 5 min, 10 min, or 15 min. After incubation, the mixture was neutralized with 500 μL of the blocking solution and titrated onto CEF and MDCK cells for NDV and AIV, respectively. A blocking solution was added to each sample before virus addition, specifically at 0 s, to confirm the neutralizing efficacy.

### Virucidal efficacy of contaminated carriers

Commercial stainless steel, rubber, and plastic sheets were prepared as small sheets measuring 5.0 × 5.0 cm and used for virucidal testing in this study. Each carrier sheet was washed with detergent and tap water for cleaning and then rinsed with dW2 to remove the detergent. Subsequently, all sheets were sterilized in an autoclave at 121°C for 15 min, dried, and stored in an incubator at 60°C until testing. During virucidal testing, each sterile carrier sheet was placed individually in a 90-mm Petri dish under a level 2 biological safety cabinet. Then 100 μL of AIV containing 5% FBS was inoculated onto the surface of each carrier. Subsequently, the virus was spread and stored inside the biological safety cabinet for 3 min. These artificially AIV-contaminated carriers were tested for virus inactivation using 4 concentrations of PPMS and compared with quaternary ammonium compounds (QAC; Bestaquam-S^®^, China Bestar Laboratories Ltd., Taiwan) at the manufacturer’s recommended concentrations. Subsequently, 500 μL of each concentration was added to each type of contaminated surface carrier and incubated for 30 s, 1 min, and 3 min. After determining the incubation period, each tested sample was neutralized to stop virucidal activity by placing the carrier in a stomacher bag containing 2 mL of the blocking solution. Subsequently, virus recovery was performed on each carrier surface by vigorously rubbing it by hand over the bag and scraping it with a sterile pipette tip to remove the virus from the carrier surfaces into the blocking solution. The resulting solution was then transferred from the stomacher bag into a microtube and diluted 10-fold using Dulbecco’s Modified Eagle Medium (DMEM, Life Technologies Corporation, NY, USA) containing 20% sodium bicarbonate, penicillin 100 units/mL, streptomycin 100 μg/mL, and amphotericin B 0.5 μg/mL, and cultured on MDCK for AIV titration. As a negative control, 500 μL of dW2 was added to each contaminated carrier, kept for 3 min, and then placed in a stomacher bag containing a blocking solution for virus removal and titration.

### Virucidal efficacy in artificially contaminated clothes

Double-fold rayon sheets (2 × 2 cm) were sterilized by autoclaving at 121°C for 15 min and then dried in an incubator at 60°C before testing, ensuring a clean experimental setup. To assess the PPMS efficacy on clothes, 100 μL of AIV mixed with 50 μL of FBS contaminated with 33% organic material was used to simulate realistic conditions. The mixture was evenly applied to the rayon sheets and allowed to air dry for 30 min at room temperature in a biosafety laboratory cabinet class II, facilitating virus adhesion. Following drying, the virus-contaminated sheets were placed into microtubes containing 500 μL of each PPMS concentration or phosphate buffer saline (PBS, pH 7.4) as negative controls. They were then incubated for specified durations of 5 s, 30 s, 1 min, 5 min, 10 min, and 15 min to evaluate the effectiveness of PPMS. After each incubation, neutralization with blocking solution halted viral activity, followed by virus recovery in MDCK cells for titration, ensuring an accurate assessment of PPMS virucidal properties. This process was repeated with sodium dichloroisocyanurate (NaDCC; Lifegard-T^®^, KBNP Inc, Chungcheongnam, South Korea) and QAC (Bestaquam) at their respective manufacturers’ recommended concentrations to compare efficacy across different disinfectants.

### Virus titration and calculation

Each NDV- and AIV-treated sample was serially diluted 10-fold using DMEM and then inoculated into CEF or MDCK cells, respectively. Before inoculation, DMEM was prepared by adding trypsin (Trypsin, Sigma, St. Louis, MO, USA) to a final concentration of 1.0 μg/mL. Following inoculation, all tissue culture plates were incubated at 37°C in a 5% carbon dioxide incubator and observed twice daily for a cytopathic effect over 3 days. Three days after inoculation, the hemagglutinin activity of the culture supernatant was detected using 1% chicken erythrocytes. Finally, the 50% tissue culture infectious dose (TCID_50_/mL) was determined using the Behrens-Kärber method [20]. Each treatment was tested in triplicate, and the titers are presented as the mean and standard error (SE).

### Inactivation analysis

The reduction factor (RF) was used to determine virus inactivation. The RF was calculated using the equation: RF = t_pc_−t_a_, where t_pc_ denotes the titer converted into a log_10_ index of the positive or PBS control, and t_a_ denotes the titer converted into an index a log_10_ index of the recovered virus from the treated sample. Virus inactivation was considered effective when the RF was >3, as demonstrated in previous studies by Takehara *et al*. [20], Thammakarn *et al*. [21], and Taesuji *et al*. [22].

### Statistical analysis

RF values were analyzed independently and presented as mean ± SE, with statistical significance determined using a one-way analysis of variance *post hoc* test (SPSS version 27, SPSS IBM, Armonk, NY, USA) between the control and treatment groups. Statistical significance was defined as p < 0.05 [9].

## Results

### pH before and after neutralization

The pH values of PPMS at 1×, 0.5×, 0.25×, and 0.125× before and after neutralization using the blocking solution are shown in Table-1.

**Table-1:** pH (mean with standard error) of potassium peroxymonosulfate before and after neutralization with blocking solution.

Concentration	Before neutralization		After neutralization	
	
	Absence of organic material	Presence of organic material	Absence of organic material	Presence of organic material
1×^a^	2.23 ± 0.01	2.31 ± 0.03	7.46 ± 0.01	7.49 ± 0.01
0.5×^b^	2.28 ± 0.20	2.60 ± 0.02	7.60 ± 0.01	7.62 ± 0.01
0.25×^c^	2.63 ± 0.01	2.97 ± 0.07	7.65 ± 0.01	7.67 ± 0.01
0.125×^d^	2.85 ± 0.01	3.58 ± 0.12	7.68 ± 0.01	7.68 ± 0.01

a Manufacturer’s recommended concentration of 1%

b Half of the recommended concentration of 0.5%

c 1/4 of the recommended concentration of 0.25%.

d 1/8 of the recommended concentration of 0.125%

### Virucidal efficacy in aqueous phase

As shown in Table-2, concentrations of 1×, 0.5×, 0.25×, and 0.125× inactivated NDV (RF ≥3 log_10_) in the absence of organic materials were within 5 s, 5 s, 5 min, and 15 min, respectively. In the presence of organic material contamination, inactivation occurred within 30 s, 1 min, and 10 min for 1×, 0.5×, and 0.25×, respectively, whereas 0.125× PPMS did not achieve inactivation within 15 min. In addition, exposure times to achieve inactivation to an undetectable level (≤2.5 log_10_ TCID_50_/mL) without organic material were 5 s and 30 s for 1× and 0.5× concentrations, respectively, while with organic material contamination, exposure times were 30 s and 10 min, respectively.

**Table-2:** Log_10_ TCID_50_/mL (mean ± standard error) of Newcastle disease virus treated with potassium peroxymonosulfate in the absence or presence of organic material.

Concentration	1×		0.5×		0.25×		0.125	
			
	Absence^a^	Presence^b^	Absence	Presence	Absence	Presence	Absence	Presence
t_pc_^c^	8.42 ± 0.38	8.42 ± 0.38	8.33 ± 0.14	8.33 ± 0.14	8.58 ± 0.14	8.58 ± 0.14	8.75 ± 0.00	8.75 ± 0.00
0 s^d^	7.83 ± 0.72	8.00 ± 0.43	8.17 ± 0.14	7.92 ± 0.14	7.83 ± 0.52	7.92 ± 0.38	8.50 ± 0.25	8.83 ± 0.14
5 s^e^	≤2.50 ± 0.00**	6.33 ± 0.29	5.00 ± 0.25*	7.42 ± 0.38	6.75 ± 0.43	7.17 ± 0.14	NT	NT
30 s	≤2.50 ± 0.00**	≤2.50 ± 0.00**	≤2.50 ± 0.00**	5.83 ± 0.29	6.42 ± 0.52	6.67 ± 0.14	NT	NT
1 min	NT	≤2.50 ± 0.00**	≤2.50 ± 0.00**	4.58 ± 0.14*	5.67 ± 0.52	6.75 ± 0.25	NT	NT
5 min	NT	NT	≤2.50 ± 0.00**	2.83 ± 0.58*	2.92 ± 0.72*	5.75 ± 0.00	6.42 ± 0.14	7.42 ± 0.52
10 min	NT	NT	≤2.50 ± 0.00**	<2.50 ± 0.00**	3.33 ± 1.04*	4.83 ± 0.14*	6.33 ± 0.63	7.33 ± 0.38
15 min	NT	NT	≤2.50 ± 0.00**	<2.50 ± 0.00**	2.83 ± 0.58*	4.17 ± 0.29*	5.50 ± 0.66*	7.25 ± 0.25

a Absence of organic material.

b Presence of organic materials.

c Titer converted into an index in log_10_ of virus control.

d Blocking solution added before NDV.

e Titer converted into an index in log_10_ of the recovered virus after indicated duration of treatment, such as 5 s, 30 s, 1 min, 5 min 10 min, and 15 min.

* Single asterisk indicates effective viral reduction (≥3 log10 TCID_50_/mL),

** Virus titer ≤2.50 log10 TCID_50_/mL indicates undetectable viral reduction. Both effective and undetectable levels of virus reduction were significantly different (p < 0.05) from virus control. TCID_50_=50% tissue culture infectious dose, NT=Not tested

Table-3 presents the virucidal activity of PPMS against AIV in the absence or presence of organic materials. Concentrations at 1×, 0.5×, 0.25×, and 0.125× of PPMS inactivated AIV within 5 s, 5 s, 5 s, and 30 s in both the absence and presence of organic materials. Moreover, inactivation to an undetectable level in the absence of organic material occurred within 5 s, 5 s, 5 s, and 30 s, respectively; in the presence of organic materials, it occurred within 5 s, 5 s, 5 s, and 5 min, respectively.

**Table-3:** Log_10_ TCID_50_/mL (mean ± standard error) of avian influenza virus treated with potassium peroxymonosulfate in the absence or presence of organic material.

C oncentration	15.45 pt		0.5×		0.25×		0.125	
			
	Absence^a^	Presence^b^	Absence	Presence	Absence	Presence	Absence	Presence
t_pc_^c^	6.83 ± 0.38	6.83 ± 0.38	6.83 ± 0.38	6.83 ± 0.38	7.33 ± 0.14	7.33 ± 0.14	7.33 ± 0.14	7.33 ± 0.14
0 s^d^	6.42 ± 0.14	6.50 ± 0.50	6.75 ± 0.66	6.83 ± 0.58	7.33 ± 0.29	7.25 ± 0.25	7.42 ± 0.29	7.00 ± 0.25
5 s^e^	≤2.50 ± 0.00**	≤2.50 ± 0.00**	≤2.50 ± 0.00**	≤2.50 ± 0.00**	≤2.50 ± 0.00**	≤2.50 ± 0.00**	4.58 ± 1.81	6.67 ± 0.38
30 s	≤2.50 ± 0.00**	≤2.50 ± 0.00**	≤2.50 ± 0.00**	≤2.50 ± 0.00**	≤2.50 ± 0.00**	≤2.50 ± 0.00**	≤2.50 ± 0.00**	3.67 ± 1.42*
1 min	NT	≤2.50 ± 0.00**	NT	≤2.50 ± 0.00**	≤2.50 ± 0.00**	≤2.50 ± 0.00**	≤2.50 ± 0.00**	3.25 ± 1.30*
5 min	NT	NT	NT	NT	NT	NT	<2.50 ± 0.00**	<2.50 ± 0.00**

a Absence of organic material.

b Presence of organic materials.

c Titer converted into an index in log_10_ of virus control.

d Blocking solution added before avian influenza virus. eTiter converted into an index in log10 of the recovered virus after indicated duration of treatment, such as 5 s, 30 s, 1 min, and 5 min.

* Single asterisk indicates effective viral reduction (≥3 log_10_ TCID_50_/mL),

** Virus titer ≤2.50 log10 TCID50/mL indicates undetectable viral reduction. Both effective and undetectable levels of virus reduction were significantly different (p < 0.05) from virus control. TCID_50_=50% tissue culture infectious dose, NT=Not tested

### Virucidal efficacy of contaminated carriers

As shown in Table-4, the virus titer recovered from AIV-contaminated carriers was presented in the range of dW2 control as 5.50–6.08 log10 TCID_50_/ml from all carriers. Concentrations of 1× PPMS could inactivate AIV on all carriers within 30 s. Together, PPMS at 0.5× and 0.25× could inactivate AIV on rubber and plastic within 30 s, whereas inactivation took place within 1 min on stainless steel. However, 0.125× PPMS and 1× QAC inactivation could not occur within 3 min in all carriers.

**Table-4:** The virucidal efficacies of PPMS at the manufacturing’s recommended concentration (1×), 0.5×, 0.25×, and 0.125×compared with the manufacturing’s recommended concentration of QAC toward the avian influenza virus on various surface carriers.

Disinfectant	Carrier type	Concentration	dW2 control (log_10_ TCID_50_/mL)	Virus titers at different contact times (TCID_50_/mL)		
	
			3 min	30 sec	1 min	3 min
PPMS	Stainless steel	1×	5.92 ± 0.38^a^	2.83 ± 0.58*	2.75 ± 0.43*	≤2.50 ± 0.00**
		0.5×	5.58 ± 0.25	2.92 ± 0.72	≤2.50 ± 0.00**	≤2.50 ± 0.00**
		0.25×	5.83 ± 0.14	4.42 ± 0.80	2.83 ± 0.58*	2.75 ± 0.43*
		0.125×	5.67 ± 0.14	4.17 ± 0.38	3.83 ± 0.63	2.92 ± 0.72
	Rubber	1×	6.00 ± 0.50	≤2.50 ± 0.00**	≤2.50 ± 0.00**	≤2.50 ± 0.00**
		0.5×	5.58 ± 0.29	≤2.50 ± 0.00**	≤2.50 ± 0.00**	≤2.50 ± 0.00**
		0.25×	6.08 ± 0.14	2.83 ± 0.38*	2.58 ± 0.14*	2.83 ± 0.58*
		0.125×	5.50 ± 0.00	4.00 ± 0.66	3.58 ± 0.88	2.92 ± 0.72
	Plastic	1×	6.00 ± 0.43	≤2.50 ± 0.00**	≤2.50 ± 0.00**	≤2.50 ± 0.00**
		0.5×	6.00 ± 0.38	<2.50 ± 0.00**	≤2.50 ± 0.00**	≤2.50 ± 0.00**
		0.25×	6.08 ± 0.52	3.08 ± 1.01*	≤2.50 ± 0.00**	≤2.50 ± 0.00**
		0.125×	5.67 ± 0.29	3.92 ± 0.72	4.00 ± 0.00	3.17 ± 0.58
QAC	Stainless steel	1×	5.75 ± 0.50	4.42 ± 0.80	4.58 ± 0.29	4.08 ± 0.63
	Rubber	1×	5.50 ± 0.25	4.50 ± 0.87	4.33 ± 0.52	3.83 ± 0.52
	Plastic	1×	5.75 ± 0.00	4.92 ± 0.80	4.58 ± 0.52	4.42 ± 0.80

a Log_10_ TCID_50_/mL (mean ± standard error) of avian influenza virus.

* Single asterisk indicates effective virus reduction (≥3 log_10_ TCID50/mL) and virus reductions are significantly different (p < 0.05) from control.

** Visible virus titer and virus reductions are significantly different (p < 0.05) from control. dW2=Distilled water, TCID_50_=50% tissue culture infectious dose, PPMS=Potassium peroxymonosulfate, NaDCC=Sodium dichloroisocyanurate, QAC=Quaternary ammonium compound

### Virucidal efficacy in artificially contaminated clothes

The effectiveness of PPMS and the manufacturer’s recommended concentrations of NaDCC and QAC against AIV were compared on infected rayon sheets contaminated with 33% organic materials. The results are presented in Table-5. PPMS at concentrations of 1×, 0.5×, 0.25×, and 0.125× inactivated AIV on a rayon sheet within 5 s, 30 s, 5 min, and 15 min, respectively. However, the recommended NaDCC concentration achieved inactivation within 10 min, whereas QAC did not achieve inactivation within 15 min. Moreover, PPMS was inactivated to an undetectable level within 30 s, 30 s, and 5 min for 1×, 0.5×, and 0.25× concentrations, respectively. However, NaDCC and QAC did not inactivate the device to an undetectable level within 15 min.

**Table-5:** Log_10_ TCID_50_/mL (mean ± standard error) of avian influenza virus following treatment on infected rayon sheets with PPMS at manufacturing-recommended concentrations (1×), 0.5×, 0.25×, and 0.125×compared with manufacturing’s recommended concentration of NaDCC and QAC even when the virus was contaminated with 33% of organic materials.

Disinfectant	Concentration	t_pc_^a^	0 s^b^	5 s^c^	30 sec	1 min	5 min	10 min	15 min
PPMS	1×	6.75 ± 0.25	6.42 ± 0.14	2.83 ± 0.58*	≤2.50 ± 0.00**	NT	NT	NT	NT
	0.5×	6.75 ± 0.25	6.67 ± 0.52	3.83 ± 2.31	≤2.50 ± 0.00**	NT	NT	NT	NT
	0.25×	6.92 ± 0.14	6.50 ± 0.43	5.92 ± 0.58	5.75 ± 0.25	5.83 ± 0.14	≤2.50 ± 0.00**	≤2.50 ± 0.00**	≤2.50 ± 0.00**
	0.125×	6.75 ± 0.25	6.50 ± 0.25	6.00 ± 0.43	5.42 ± 0.88	5.67 ± 0.76	4.25 ± 1.64	3.92 ± 1.66	2.83 ± 0.58*
NaDCC	1×	6.92 ± 0.14	6.67 ± 0.29	6.08 ± 0.14	5.83 ± 0.38	5.42 ± 0.80	4.83 ± 0.58	3.83 ± 0.58*	2.83 ± 0.58*
QAC	1×	7.42 ± 0.14	6.83 ± 0.29	NT	NT	NT	6.42 ± 0.14	6.33 ± 0.52	5.58 ± 1.26

a Titer converted into an index in log_10_ of virus control.

b Blocking solution added before infected rayon sheet.

c Titer converted into an index in log_10_ of recovered virus after indicated duration of treatment, such as 5 s, 30 s, 1 min, 5 min 10 min, and 15 min.

* Single asterisk indicates effective virus reduction (≥3 log_10_ TCID_50_/mL) and virus reductions are significantly different (p < 0.05) from control.

** Visible virus titer and virus reductions are significantly different (p < 0.05) from control. TCID_50_=50% tissue culture infectious dose, PPMS=Potassium peroxymonosulfate, NaDCC=Sodium dichloroisocyanurate, QAC=Quaternary ammonium compound, NT=Not tested

## Discussion

The PPMS mechanism exhibits high acidity (pH 2.23–2.85), and after neutralization with a blocking solution, the pH becomes neutral (pH 7.46–7.68). In the present study, all concentrations of PPMS at 0 s did not inactivate NDV or AIV, and the titers did not differ from those of the virus control (t_pc_). These findings suggest that the potent acidity of PPMS is the primary mechanism by which it eliminates viruses. Moreover, the efficacy of the blocking solution may affect the inhibitory activity of PPMS.

PPMS is a potassium salt of peroxymonosulfuric acid, widely used as an oxidizing agent. Sonthipet *et al*. [6] and Kunanusont *et al*. [9] have described PPMS as synonymous with PMPS, which acts on bacteria and viruses by oxidation and is especially active against capsid protein, destroying the nucleic acids of the viruses. Our results demonstrate aqueous phase testing at optimal concentrations, organic material conditions, and exposure timing. In addition, inactivation of NDV to undetectable levels required an extended exposure time to ensure the inactivation of all viruses. However, AIV inactivation did not require an extended time, except at 0.125×. A comparison of the virucidal efficacy between NDV and AIV indicated that AIV was more susceptible to PPMS inactivation. These results concur with Ruenphet *et al*. [17, 18], who reported that fresh charcoal ash, slaked lime, and food additive grade calcium hydroxide inactivated AIV more easily than NDV.

The present study evaluated the efficacy of aqueous disinfectants. The study examined their application on artificially contaminated carrier surfaces and clothes, comparing the efficacy of PPMS with NaDCC and QAC. In general, pathogens such as bacteria and viruses are excreted with organic materials or cell debris from infected animals, adhering firmly to surface equipment around animals [23]. The present results indicate that PPMS can inactivate viruses on all surface carriers at the recommended concentrations within the shortest exposure time (30 s). It also inactivated AIV on rubber and plastic within 30 s and on stainless steel within 1 min, even at one-fourth of the manufacturer’s recommended PPMS concentration. However, the virucidal efficacy of QAC did not affect all carriers at the manufacturer’s recommended concentration. These carrier models are commonly found in vehicle tires, boots, tracks, and various animal farm equipment, including feeders, water pots, egg trays, and chicken transport cages. Thus, this study confirms the efficacy and suitability of PPMS for inactivating AIV on all carrier surfaces commonly found near animal farms.

Our model simulated AIV artificially contaminated with organic materials on rayon sheets, mimicking carpets, beddings, towels, or clothes. In this study, NaDCC and QAC, which are marketed as effective against most bacterial and viral pathogens, were tested on rayon. The efficacy of QAC was eva- luated and compared with that of PPMS. QAC is a cationic detergent, and NaDCC is a slow-release chlorine source commonly used in disinfecting animal farms and food processing industries such as car washes, foot baths, and slaughterhouses. QAC offers the advantage of low toxicity and a broad antimicrobial spectrum. However, its inactivation efficacy is often reduced even in the presence of organic material contamination [24, 25]. As shown in Table-4, 1/8 of the manufacturer’s recommended concentration (0.125×) of PPMS inactivated the virus inside the rayon sheets within 15 min. However, at higher concentrations (1×, 0.5×, and 0.25×), PPMS achieved faster AIV destruction than 0.125×. The manufacturer’s recommended concentration (1×) for NaDCC required an extended exposure time of 10 min, whereas QAC required more than 15 min. The results indicated that PPMS had higher efficacy than NaDCC and QAC, suggesting its utility as an alternative disinfectant or virucidal agent to inactivate AIV on contaminated carpets, clothes, towels, and bedding, especially in animal farms or hospitals.

In this study, PPMS was effective against enve- loped viruses on diverse carrier surfaces and clothing materials. However, the present study was confined to *in vitro* tests and did not directly include *in vivo* or infectivity trials involving animals. Nevertheless, findings from *in vivo* assessments may exhibit notable discrepancies compared to those derived from *in vitro* experiments.

## Conclusion

The results of the present study indicate that PPMS can inactivate enveloped viruses such as NDV and AIV. Moreover, its efficacy on various carrier surfaces and artificially contaminated clothes is superior to NaDCC and QAC. However, virucidal efficacy depends on the optimal concentration, organic material conditions, and exposure/contact timing. Therefore, PPMS should be used as an alternative disinfectant to enhance biosecurity to control viruses that contaminate animal farms, slaughterhouses, or hospitals.

## Authors’ Contributions

SR, NK, and DP: Contributed to the study conception, designed, conducted the experiments, and analyzed the data. SR and NK: Contributed to sample preparation. SR and DP: Drafted the manuscript. All authors have read and approved the final manuscript.
